# Oral Health Among Medicare Beneficiaries in Nursing Homes

**DOI:** 10.1001/jamanetworkopen.2023.33367

**Published:** 2023-09-12

**Authors:** Steffany Chamut, Carla Shoff, Kristiana Yao, Lee A. Fleisher, Natalia I. Chalmers

**Affiliations:** 1 Oral Health Policy and Epidemiology, Harvard School of Dental Medicine, Boston, Massachusetts; 2 Office of the Administrator, Centers for Medicare & Medicaid Services, Baltimore, Maryland; 3 Center for Clinical Standards and Quality, Centers for Medicare & Medicaid Services, Baltimore, Maryland

## Abstract

This cross-sectional study investigates rates of dental problems among Medicare beneficiaries in nursing homes and characteristics associated with these rates.

## Introduction

Older adults in the US encounter significant barriers to maintaining good oral health, and disparities in disease prevalence and access to oral care are persistent challenges.^1^ Notably, a substantial portion of Medicare beneficiaries (51%) lack dental coverage, further exacerbating the problem.^2^ This study’s primary goals were to describe the prevalence of dental problems among Medicare beneficiaries residing in nursing homes and identify characteristics associated with experiencing dental problems.

## Methods

This cross-sectional study included Medicare beneficiaries residing in Centers for Medicare & Medicaid Services (CMS)–certified nursing homes in 2020. We used the CMS Minimum Data Set 3.0 Resident Assessment Instrument data^3^ linked to the 2020 Medicare Beneficiary Summary File Base.^4^ Race and ethnicity were self-reported to the Social Security Administration and integrated with enrollment in Medicare. We used χ^2^ tests to test differences across groups, with statistical significance set at a 2-tailed *P* < .05. We used 6 multilevel logistic regression models to estimate odds of experiencing each dental problem (eMethods in Supplement 1). Reporting of this study followed the STROBE reporting guideline, was covered by the Common Rule exemption 45 CFR §46.104(d)(4)(iv), and did not require institutional review board review.

## Results

The study sample comprised 2 355 366 Medicare beneficiaries (2 142 384 aged ≥65 years [91.0%]; 1 446 969 females [61.4%]; 303 738 non-Hispanic Black [12.9%], 141 383 Hispanic [6.0%], and 1 827 593 non-Hispanic White [77.6%]) (Table 1). The most prevalent dental problem per 1000 beneficiaries was no natural teeth or tooth fragments (175.10 beneficiaries), followed by cavities or broken natural teeth (72.89 beneficiaries); pain, discomfort, or difficulty chewing (10.79 beneficiaries); broken or loosely fitting dentures (9.61 beneficiaries); inflamed or bleeding gums or loose teeth (2.15 beneficiaries); and abnormal mouth tissue (2.06 beneficiaries).

**Table 1.** Study Population Characteristics and Dental Problem Prevalence

Characteristic	Total study population	Broken or loosely fitting dentures		No natural teeth or tooth fragments		Abnormal mouth tissue (ulcers, masses, or lesions)		Cavity or broken natural teeth		Inflamed or bleeding gums or loose teeth		Pain, discomfort, or difficulty chewing	
	No. (rate)^a^	No. (rate)^a^	*P* value^b^	No. (rate)^a^	*P* value^b^	No. (rate)^a^	*P* value^b^	No. (rate)^a^	*P* value^b^	No. (rate)^a^	*P* value^b^	No. (rate)^a^	*P* value^b^
Overall	2 355 366 (1000)	22 631 (9.61)	NA	412434 (175.1)	NA	4858 (2.06)	NA	171 677 (72.89)	NA	5074 (2.15)	NA	25419 (10.79)	NA
Age group, y													
<65	212 982 (90.42)	1355 (6.36)	<.001	26 963 (126.60)	<.001	395 (1.85)	.09	22 753 (106.83)	<.001	701 (3.29)	<.001	2091 (9.82)	<.001
65-74	545 910 (231.77)	4341 (7.95)		90 273 (165.36)		1157 (2.12)		45 998 (84.26)		1274 (2.33)		5583 (10.23)	
75-84	736 225 (312.57)	7017 (9.53)		135 361 (183.86)		1555 (2.11)		47 788 (64.91)		1402 (1.90)		7916 (10.75)	
≥85	860 249 (365.23)	9918 (11.53)		159 837 (185.80)		1751 (2.04)		55 138 (64.10)		1697 (1.97)		9829 (11.43)	
Sex													
Female	1 446 969 (614.33)	14166 (9.79)	<.001	256 213 (177.07)	<.001	3064 (2.12)	.02	93 403 (64.55)	<.001	3059 (2.11)	.09	15873 (10.97)	<.001
Male	908 397 (385.67)	8465 (9.32)		156 221 (171.97)		1794 (1.97)		78 274 (86.17)		2015 (2.22)		9546 (10.51)	
Race and ethnicity^c^													
American Indian or Alaskan Native	11 596 (4.92)	154 (13.28)	<.001	2911 (251.03)	<.001	30 (2.59)	<.001	1210 (104.35)	<.001	43 (3.71)	<.001	173 (14.92)	<.001
Asian or Pacific Islander	44 320 (18.82)	296 (6.68)		6901 (155.71)		118 (2.66)		2933 (66.18)		126 (2.84)		407 (9.18)	
Black	303 738 (128.96)	2196 (7.23)		55 415 (182.44)		424 (1.40)		25 773 (84.85)		687 (2.26)		2564 (8.44)	
Hispanic	141 383 (60.03)	1127 (7.97)		20 818 (147.25)		204 (1.44)		9777 (69.15)		298 (2.11)		1547 (10.94)	
White	1 827 593 (775.93)	18658 (10.21)		322 983 (176.73)		4021 (2.20)		130 021 (71.14)		3849 (2.11)		20460 (11.20)	
Other^d^	26 736 (11.35)	200 (7.48)		3406 (127.39)		61 (2.28)		1963 (73.42)		71 (2.66)		268 (10.02)	
Medicare program													
Fee for service	1 446 584 (614.17)	13972 (9.66)	.32	252 577 (174.60)	.01	3035 (2.10)	.13	107 191 (74.10)	<.001	3225 (2.23)	.002	15765 (10.90)	.047
Medicare Advantage	908 782 (385.83)	8659 (9.53)		159 857 (175.90)		1823 (2.01)		64 486 (70.96)		1849 (2.03)		9654 (10.62)	
Dual eligibility status													
Medicare and Medicaid	1 283 006 (544.72)	12796 (9.97)	<.001	276 180 (215.26)	<.001	2364 (1.84)	<.001	114 329 (89.11)	<.001	3493 (2.72)	<.001	13117 (10.22)	<.001
Medicare only	1 072 360 (455.28)	9835 (9.17)		136 254 (127.06)		2494 (2.33)		57 348 (53.48)		1581 (1.47)		12302 (11.47)	
Alzheimer disease or dementia													
Diagnosed	845 722 (359.06)	8135 (9.62)	.90	168 356 (199.07)	<.001	1387 (1.64)	<.001	70 463 (83.32)	<.001	2161 (2.56)	<.001	8221 (9.72)	<.001
Not diagnosed	1 509 644 (640.94)	14496 (9.60)		244 078 (161.68)		3471 (2.30)		10 1214 (67.04)		2913 (1.93)		17 198 (11.39)	
Chronic medical conditions, No.													
0	266 941 (113.33)	2260 (8.47)	<.001	37 664 (141.09)	<.001	447 (1.67)	<.001	21 922 (82.12)	<.001	781 (2.93)	<.001	2567 (9.62)	<.001
1	891 551 (378.52)	7849 (8.80)		139 397 (156.35)		1534 (1.72)		65 530 (73.50)		1920 (2.15)		8904 (9.99)	
2	702 627 (298.31)	6973 (9.92)		128 705 (183.18)		1566 (2.23)		49 714 (70.75)		1440 (2.05)		7696 (10.95)	
≥3	494 247 (209.84)	5549 (11.23)		106 668 (215.82)		1311 (2.65)		34 511 (69.83)		933 (1.89)		6252 (12.65)	
Mental health disorders, No.													
0	1 101 291 (467.57)	9999 (9.08)	<.001	176 550 (160.31)	<.001	2247 (2.04)	.78	73 176 (66.45)	<.001	2122 (1.93)	<.001	11314 (10.27)	<.001
1	726 237 (308.33)	7278 (10.02)		129 807 (178.74)		1515 (2.09)		54 009 (74.37)		1614 (2.22)		7997 (11.01)	
≥2	527 838 (224.10)	5354 (10.14)		106 077 (200.97)		1096 (2.08)		44 492 (84.29)		1338 (2.53)		6108 (11.57)	
Designation of nursing home													
Urban	1 923 805 (816.78)	16582 (8.62)	<.001	306 268 (159.20)	<.001	3765 (1.96)	<.001	129 070 (67.09)	<.001	3935 (2.05)	<.001	19 984 (10.39)	<.001
Rural	431 561 (183.22)	6049 (14.02)		106 166 (246.00)		1093 (2.53)		42 607 (98.73)		1139 (2.64)		5435 (12.59)	

Abbreviation: NA, not applicable.

^a^ Rate is per 1000 Medicare beneficiaries in nursing homes.

^b^ χ^2^ tests were used to test statistical significance.

^c^ Race groups include individuals with that race who were not Hispanic. The same race and ethnicity categories self-reported to the Social Security Administration are reported in this study. Race and ethnicity were assessed due to significant disparities in the prevalence and severity of oral diseases and access to dental services.

^d^ Other race includes unknown or other race.

Significant differences in dental problem prevalence were observed across demographic and clinical groups (Table 2). Non-Hispanic Black beneficiaries had 16% higher odds of having no natural teeth or tooth fragments (adjusted odds ratio [OR], 1.16; 95% CI, 1.15-1.18) and 5% higher odds of having cavities or broken natural teeth (aOR, 1.05; 95% CI, 1.03-1.07) compared with White beneficiaries. Similarly, American Indian or Alaskan Native beneficiaries had 34% higher odds of having no natural teeth or tooth fragments (aOR, 1.34; 95% CI, 1.27-1.40), 20% higher odds of having cavities or broken natural teeth (aOR, 1.20; 95% CI, 1.11-1.29), and 45% higher odds of having inflamed or bleeding gums or loose teeth (aOR, 1.45; 95%, 1.05-2.01) compared with White beneficiaries. Beneficiaries with 3 or more chronic conditions had increased odds of having broken or loosely fitting dentures (aOR, 1.26; 95% CI, 1.20-1.33), no natural teeth or tooth fragments (aOR, 1.57; 95% CI, 1.54-1.59), abnormal mouth tissue (aOR, 1.50; 95% CI, 1.34-1.67), and pain, discomfort, or difficulty chewing (aOR, 1.22; 95% CI, 1.16, 1.28) compared with beneficiaries with no chronic conditions. However, they had 16% lower odds of having cavities or broken natural teeth (aOR, 0.84; 95% CI, 0.83-0.86) and 27% lower odds of having inflamed or bleeding gums or loose teeth (aOR, 0.73; 95% CI, 0.66-0.81) compared with beneficiaries with no chronic conditions. Additionally, beneficiaries in rural nursing homes were more than 70% more likely to experience 3 of 6 dental problems and more than 30% more likely to experience the other 3 dental problems than beneficiaries in urban nursing homes.

**Table 2.** Odds of Experiencing Dental Problems

Characteristic	Dental problem, aOR (95% CI) (N = 2 355 366)^a^					
	Broken or loosely fitting dentures	No natural teeth or tooth fragments	Abnormal mouth tissue (ulcers, masses, or lesions)	Cavity or broken natural teeth	Inflamed or bleeding gums or loose teeth	Pain, discomfort, or difficulty chewing
Age group, y						
<65	0.50 (0.47-0.53)	0.43 (0.42-0.44)	1.03 (0.92-1.17)	1.39 (1.36-1.42)	1.60 (1.44-1.78)	0.89 (0.84-0.94)
65-74	0.65 (0.63-0.68)	0.70 (0.70-0.71)	1.09 (1.00-1.18)	1.21 (1.19-1.22)	1.26 (1.16-1.36)	0.88 (0.85-0.92)
75-84	0.81 (0.78-0.83)	0.89 (0.89-0.90)	1.05 (0.98-1.13)	0.96 (0.95-0.98)	1.03 (0.95-1.11)	0.91 (0.88-0.94)
≤85	1 [Reference]	1 [Reference]	1 [Reference]	1 [Reference]	1 [Reference]	1 [Reference]
Sex						
Female	0.95 (0.92-0.98)	0.95 (0.94-0.96)	1.09 (1.03-1.16)	0.74 (0.74-0.75)	0.95 (0.90-1.01)	0.99 (0.96-1.01)
Male	1 [Reference]	1 [Reference]	1 [Reference]	1 [Reference]	1 [Reference]	1 [Reference]
Race and ethnicity^b^						
American Indian or Alaskan Native	1.03 (0.86-1.22)	1.34 (1.27-1.40)	1.06 (0.73-1.54)	1.20 (1.11-1.29)	1.45 (1.05-2.01)	1.11 (0.93-1.31)
Asian or Pacific Islander	0.87 (0.76-0.98)	1.07 (1.04-1.10)	0.94 (0.74-1.19)	0.98 (0.93-1.02)	1.06 (0.85-1.33)	1.21 (1.08-1.35)
Black	0.92 (0.88-0.97)	1.16 (1.15-1.18)	0.76 (0.69-0.85)	1.05 (1.03-1.07)	1.04 (0.94-1.14)	1.02 (0.97-1.07)
Hispanic	0.90 (0.84-0.97)	0.98 (0.96-1.00)	0.81 (0.69-0.94)	0.96 (0.94-0.99)	1.00 (0.88-1.14)	1.03 (0.97-1.10)
White	1 [Reference]	1 [Reference]	1 [Reference]	1 [Reference]	1 [Reference]	1 [Reference]
Other race^c^	0.93 (0.81-1.07)	0.83 (0.80-0.87)	1.04 (0.800-1.34)	0.97 (0.92-1.02)	1.09 (0.86-1.40)	1.06 (0.94-1.21)
Medicare program						
Fee for service	0.94 (0.91-0.97)	0.96 (0.95-0.96)	1.01 (0.95-1.07)	1.01 (1.00-1.02)	0.99 (0.93-1.06)	1.01 (0.98-1.03)
Medicare Advantage	1 [Reference]	1 [Reference]	1 [Reference]	1 [Reference]	1 [Reference]	1 [Reference]
Dual eligibility status						
Medicare and Medicaid	1.23 (1.20-1.27)	1.79 (1.77-1.80)	0.86 (0.81-0.92)	1.29 (1.27-1.30)	1.49 (1.40-1.60)	1.07 (1.04-1.10)
Medicare only	1 [Reference]	1 [Reference]	1 [Reference]	1 [Reference]	1 [Reference]	1 [Reference]
Alzheimer disease or dementia						
Diagnosed	0.84 (0.82-0.87)	1.01 (1.00-1.01)	0.72 (0.68-0.78)	1.18 (1.17-1.20)	1.24 (1.16-1.32)	0.82 (0.80-0.85)
Not diagnosed	1 [Reference]	1 [Reference]	1 [Reference]	1 [Reference]	1 [Reference]	1 [Reference]
Chronic medical conditions, No.						
0	1 [Reference]	1 [Reference]	1 [Reference]	1 [Reference]	1 [Reference]	1 [Reference]
1	1.03 (0.98-1.08)	1.09 (1.07-1.10)	1.03 (0.93-1.15)	0.93 (0.91-0.94)	0.82 (0.75-0.89)	1.02 (0.98-1.07)
2	1.14 (1.09-1.20)	1.30 (1.29-1.32)	1.31 (1.18-1.46)	0.88 (0.86-0.90)	0.80 (0.73-0.87)	1.11 (1.06-1.16)
≥3	1.26 (1.20-1.33)	1.57 (1.54-1.59)	1.50 (1.34-1.67)	0.84 (0.83-0.86)	0.73 (0.66-0.81)	1.22 (1.16-1.28)
Mental health disorders, No.						
0	1 [Reference]	1 [Reference]	1 [Reference]	1 [Reference]	1 [Reference]	1 [Reference]
1	1.10 (1.07-1.14)	1.03 (1.02-1.04)	1.05 (0.98-1.12)	1.00 (0.98-1.01)	1.04 (0.98-1.12)	1.10 (1.06-1.13)
≥2	1.15 (1.11-1.19)	1.09 (1.08-1.11)	1.06 (0.98-1.15)	1.00 (0.99-1.01)	1.09 (1.01-1.18)	1.18 (1.14-1.22)
Designation of nursing home						
Urban	1 [Reference]	1 [Reference]	1 [Reference]	1 [Reference]	1 [Reference]	1 [Reference]
Rural	1.72 (1.63-1.83)	1.73 (1.66-1.81)	1.36 (1.24-1.49)	1.73 (1.64-1.82)	1.37 (1.23-1.53)	1.37 (1.28-1.47)

Abbreviation: aOR, adjusted odds ratio.

^a^ Models adjusted for all variables in the table, and all models were clustered at the nursing home level.

^b^ Race groups include individuals with that race who were not Hispanic.

^c^ Other race includes unknown and other race. The same race and ethnicity categories self-reported to the Social Security Administration are reported in this study. Race and ethnicity were assessed due to significant disparities in the prevalence and severity of oral diseases and access to dental services.

## Discussion

This cross-sectional study’s findings highlight the considerable prevalence of dental problems among Medicare beneficiaries in nursing homes, revealing disparities across various demographic and clinical factors. Notably, beneficiaries with more chronic conditions were more likely to be edentulous, which makes eating properly and managing other health conditions challenging.

Our study was limited by its sole focus on Medicare beneficiaries in nursing homes, which may limit the generalizability of our findings to all US long-term care populations. Additionally, the possibility of reporting bias in nursing homes cannot be ignored. Although our models accounted for beneficiary similarities and assessment uniformity, variations in how patient assessments were conducted could pose a concern and introduce bias to our findings. Targeted interventions addressing oral health disparities in this high-risk population are essential to improve overall health and well-being.

## Notes

Supplement 1.
eMethods.

Supplement 2.
Data Sharing Statement
