# Supplementary material for: Oral Health Among Medicare Beneficiaries in Nursing Homes
Source: JAMA Netw Open. 2023 Sep 12;6(9):e2333367. doi: 10.1001/jamanetworkopen.2023.33367 (PMC10498323; doi:10.1001/jamanetworkopen.2023.33367)
Supplement: Supplement 1. — eMethods. [file jamanetwopen-e2333367-s001.pdf]

Supplemental Online Content

Chamut S, Shoff C, Yao K, Fleisher LA, Chalmers NI. Oral health among Medicare beneficiaries in nursing homes. *JAMA Netw Open*. 2023;6(9):e2333367. doi:10.1001/jamanetworkopen.2023.33367

eMethods

This supplemental material has been provided by the authors to give readers additional information about their work.

© 2023 Chamut S et al. *JAMA Network Open.*

eMethods.

Minimum Data Set 3.0 Resident Assessment Instrument

The Centers for Medicare & Medicaid Services (CMS) Minimum Data Set (MDS) 3.0 Resident Assessment Instrument (RAI) is a standardized assessment used to support care planning, inform payment, and can be utilized to assess oral health in the nursing home population. The MDS 3.0 RAI includes the assessment of oral health status. Under federal regulations, a health professional with demonstrated competency (e.g., dentist, physician, or nurse), must assess a resident's oral health status within 14 days upon admission, at periodic intervals throughout the stay, and following certain events. In addition, the nursing home must provide proper arrangements for comprehensive diagnoses and dental treatments through in-house or organized dental services from qualified personnel. Prior research on oral health in long-term care or nursing home settings suggests that residents may not always receive adequate oral care.

Study Population

A cross-sectional cohort design was used, consisting of Medicare Fee-for-Service and Medicare Advantage beneficiaries who had assessments while residing in a CMS certified nursing home in 2020. CMS certified nursing homes provide skilled nursing facility or nursing facility care, and in most cases, both types of care. Beneficiaries included in this study received an assessment during a skilled nursing facility or nursing facility stay. The MDS 3.0 data were limited to assessments that were not missing a beneficiary ID or a CMS certification number (CCN), assessed a beneficiary who is enrolled in Medicare (including beneficiaries dually enrolled in Medicare and Medicaid), were performed at a nursing home with more than 10 Medicare beneficiaries in the facility and were not missing data on any of the dental measures.

© 2023 Chamut S et al. *JAMA Network Open.*

Beneficiaries with more than one assessment in 2020 (23.0%) were limited to one assessment with a series of checks with data-driven preference given using the following order (ordered by most to least likely to be selected): MDS item subset code (comprehensive, quarterly, prospective payment system); federal OBRA reason for assessment (admission comprehensive, significant change in status assessment (SCSA) comprehensive, annual comprehensive, significant correction of a prior assessment (SCPA) comprehensive, and quarterly non-comprehensive); and assessment date oldest to most recent. Only beneficiaries with complete data were included in the final analytic dataset, resulting in 2,355,366 beneficiaries (0.05% removed due to missing data).

Outcomes

There are six dental problems derived from the MDS 3.0 Section L: Oral/Dental Status that are included as outcomes in this study: 1) broken or loosely fitting full or partial denture (chipped, cracked, uncleanable, or loose); 2) no natural teeth or tooth fragment(s) (edentulous); 3) abnormal mouth tissue (ulcers, masses, oral lesions, including under denture or partial if one is worn); 4) obvious or likely cavity or broken natural teeth; 5) inflamed or bleeding gums or loose natural teeth; 6) mouth or facial pain, discomfort or difficulty with chewing.

Demographic, Enrollment, Clinical, and Skilled Nursing Facility Covariates

Beneficiary demographic and enrollment covariates, obtained from the 2020 Medicare Beneficiary Summary File Base, include age group, sex, race and ethnicity, Medicare program, and dual-eligibility status. Beneficiary clinical covariates include Alzheimer's disease or dementia diagnosis, count of chronic medical condition diagnoses (cancer; coronary artery disease; heart failure; hypertension; asthma, COPD, or chronic lung disease), and count of mental disorder diagnoses (anxiety disorder; depression; manic depression; psychotic disorder;

© 2023 Chamut S et al. *JAMA Network Open.*

schizophrenia). Nursing homes are designated as rural or urban according to the facility's location, as reported in MDS 3.0.

Statistical Analysis

Chi-square tests were used to test significant differences in dental problem prevalence rates across demographic and clinical category groups. Six multilevel logistic regression models were used to predict the odds of experiencing each dental problem. The CMS certification number was included in the models as a level-two random intercepts parameter to adjust for the uniformity of assessments and similarity of beneficiaries residing in the same nursing home. All multilevel logistic regression model results are reported as adjusted odds ratios (aOR) with the corresponding 95% confidence intervals (Wald-type). Multicollinearity was assessed for all models using the variance inflation factor (VIF). Multicollinearity was not an issue in any models, as the VIF was never above 2. All analyses were performed using SAS Enterprise Guide version 7.1 (Table 1) and Stata version 17.0 (Table 2).

© 2023 Chamut S et al. *JAMA Network Open.*
