# Supplementary material for: Oral Health Among Medicare Beneficiaries in Nursing Homes
Source: JAMA Netw Open. 2023 Sep 12;6(9):e2333367. doi: 10.1001/jamanetworkopen.2023.33367 (PMC10498323; doi:10.1001/jamanetworkopen.2023.33367)
Supplement: Supplement 2. — Data Sharing Statement [file jamanetwopen-e2333367-s002.pdf]

Data Sharing Statement

Chamut. Oral Health Among Medicare Beneficiaries in Nursing Homes. *JAMA Netw Open*. Published online September 12, 2023. doi:10.1001/jamanetworkopen.2023.33367

Data

Data available: No

Additional Information

Explanation for why data not available: Access to these data requires an active data use agreement with the Centers for Medicare & Medicaid Services.
